# Contrasting Effects of “External” Worker’s Proactive Behavior on Their Turnover Intention: A Moderated Mediation Model

**DOI:** 10.3390/bs11050070

**Published:** 2021-05-06

**Authors:** Seonjo Kim, Jun Ishikawa

**Affiliations:** 1 Faculty of Economics and Business Administration, Kanayagawa Campus, Fukushima University, Fukushima 960-1296, Japan; 2 College of Business, Rikkyo University, Tokyo 171-8501, Japan; jun@rikkyo.ac.jp

**Keywords:** portfolio career worker, proactive behavior, turnover intention, workplace ostracism, employee–organization relationship in human resource practices, status in a new organization

## Abstract

Interpersonal conflicts between portfolio career workers (hereafter, PCWs) who entered from the external labor market and existing permanent workers are a controversial workplace issue in South Korea. This study examines whether the existing permanent workers’ responses to the newcomers speaking up depend on the type of proactive behavior, that is, whether PCWs speak within extra-role or in-role boundaries. We found that PCWs perceive more workplace ostracism when they are proactive outside their job boundaries and less workplace ostracism when they are proactive inside their job boundaries. Further, their perceptions of ostracism lead to intentions of turnover. These relationships are conditional on the type of employee–organization relationship and the PCWs’ status in a new organization. Data were collected from 261 PCWs in Korea. Bootstrap-based conditional process analyses were utilized to test the hypothesized model. The results show that workplace ostracism mediates the relationship between the two types of proactive behavior and turnover intention, but in contrasting directions. The effect of the two types of proactive behavior on workplace ostracism is stronger for higher levels of reciprocal relationship between organization and employees, while the effect of workplace ostracism on turnover intention is stronger for higher levels of PCWs’ status in a new organization. Thus, the workplace conflicts PCWs face not only represent interpersonal problems within the workplace but also constitute a multilayered phenomenon related to the long-term institutionalized relationships between organizations and employees.

## 1. Introduction

This study answers the following questions: First, does the newcomer’s proactive behavior always lead to existing employees’ positive response in terms of interpersonal relations? If not, what type of proactive behavior leads to a negative response? Second, in what kind of boundary conditions would the relationship between proactive behavior and negative response be strengthened (or weakened)? Third, is the existing employees’ negative response strong enough to affect the newcomer’s turnover intention? Fourth, if so, in what kind of boundary conditions would the relationship between existing employees’ negative response and the newcomer’s turnover intention be strengthened (weakened)?

The early resignation of newly hired employees is a major concern for human resources (HR) personnel. In particular, the early resignation of portfolio career workers (hereafter “PCWs”) is very expensive for companies because the talent pool of PCWs with expertise in specific jobs is limited [1]. PCWs are hired as permanent employees on the basis of their past career track and performance [2]. Occupationally qualified, self-motivating professionals with marketable and well-defined specific skills are prioritized when recruiting PCWs.

There are fundamental differences between the public recruitment process intended for college graduates and unannounced recruitment process for experienced applicants in terms of hiring standards, recruitment period, scale, and treatment. To elaborate, here is a hypothetical scenario of Samsung Electronics in Korea expanding its semiconductor plant on a large scale that would require the hiring of research and development (R&D) personnel in the “Z-NAND” field. There are two popular ways of hiring such professionals. One is to recruit college graduates from the engineering departments of universities through the public recruitment process and foster in-house experts in the Z-NAND field with a long-term view. The other is to scout for R&D personnel in the field from competitors (e.g., SK Hynix) or hire doctorate graduates in engineering with a specialty in source technology in the same field.

First, the public recruitment process is conducted on an annually fixed schedule (usually in March and September), whereas the unannounced recruitment for experienced personnel is driven mainly by unforeseen business needs. In other words, Samsung Electronics will proceed with the public recruitment process in March and September for engineering college graduates regardless of the semiconductor plant expansion plan. However, the recruitment for experienced personnel in the Z-NAND field will not be conducted without the urgent need for the semiconductor plant expansion, and thus, it will remain unaffected by the aforementioned recruitment schedule.

Second, the public recruitment process employs numerous people adhering to an HR plan based on the quota that was set after calculating the company’s total number of retired manpower resources. However, the recruitment process for experienced personnel is conducted based on sporadic and urgent business or departmental needs, and hence, only a small number of people can be hired at a time. If Samsung Electronics has foreseen a demand in manpower for the Z-NAND field, there will be an in-house cultivation of the needed specialists with that particular area of expertise. In this respect, the recruitment process for experienced personnel is deemed an instance of special recruitment solely aimed at meeting the urgent needs that diverge from the original manpower and personnel plan. 

Third, the public recruitment process hires on the basis of an individual’s aptitude or personality, but the recruitment for experienced personnel is based on an individual’s subject matter expertise or past experience. For example, Samsung Electronics scrutinizes whether the engineering college graduates possess the necessary analytical skills or the perseverance traits that are often required for R&D personnel positions. On the other hand, the recruitment for experienced personnel is not based on abstract analytical ability or perseverance traits, but more on whether they have the necessary subject matter expertise on the Z-NAND field, the original technological capability (based on the Ph.D. dissertation topic or patent application track record), or the relevant Z-NAND field experience and achievements (based on the resume or reference check).

Finally, those hired through the public recruitment process will join the company as entry-level associates with no exception across the board, but those selected through the experience-based recruitment process will join the company with seniority, entering at the position of Senior Researcher or a higher position based on their career experience. For example, in the case of Samsung Electronics, those with a Ph.D. in engineering are hired as Senior Researchers with a guarantee of a promotion within a year or two. Additionally, if the individual has three years’ experience as a post-doctoral researcher, he/she may join the department as a grade three manager. Furthermore, most of the SK Hynix’s deputy department head-level research and development professionals are promised a befitting compensation package. If the individual has an unrivaled level of specialty, he/she may be hired at the department head level. In this process, there may be negotiations between the HR department and the individual expected to join the company with regard to the salary and the compensation package.

Recruiting and sustaining PCWs, who form the majority of senior staff members and deputy-rank strategists in their thirties or older, incur relatively high costs [3]. Moreover, when PCWs resign early, it adversely affects the reputation of the company and hinders subsequent recruitment [4]. Despite these challenges, companies’ need for PCWs, who are expected to be “industry-ready” (to have “ready fighting power”; [5], is continually increasing. The percentage of PCWs among the newly hired is estimated at about 40% [1,4], and 43.8% of companies that have adopted a recruitment system for PCWs have reported that they intend to expand it [3].

No study has investigated the interpersonal conflict between PCWs and existing employees. However, companies that have adopted a recruitment system for PCWs have found that these newcomers often complain of isolation in their interpersonal relationships with existing employees. Industry reports point out not only the absence of interpersonal relationships in a passive sense but also the problem of exclusion in a proactive sense, such as through discrimination by existing employees. For example, ref. [1] found through an interview conducted on PCWs that they perceived their company and departments implicitly classifying existing permanent employees hired through the recruitment of new graduates (hereafter, “existing permanent employees”) and PCWs. Meanwhile, ref. [4] found that existing permanent employees are concerned that PCWs may deprive them of their positions. In fact, according to [3], about 17% of HR personnel in companies that have adopted a recruitment system for PCWs identify “competition and conflict between PCWs and existing employees” as a problem associated with this new recruitment system. 

This study seeks to identify how the proactive behavior of PCWs influences turnover intention from the perspective of workplace ostracism. 

We focus on the proactive behavior because companies expect PCWs to proactively improve their business processes based on their expertise and experience accumulated during their past careers. For example, HR personnel cite “high work productivity” (34.5%) and the “enhancement of the competitiveness of existing business” (29.2%) as the main objectives of recruitment for PCWs. However, previous studies warn of the possibility of evoking feelings of opposition and conflict and point out that proactive behavior is not always embraced [6,7,8,9].

Previous studies on proactive behavior generally focused on its positive effects on job attitude, career success, and group performance [10,11,12]. That is no exception in the so-called “newcomer study”. For example, ref. [13] reported a positive relationship between proactive behavior and socialization outcomes, such as social integration, job satisfaction, organizational commitment, and intention to remain. 

However, recent studies pointed out that proactivity has pros as well as cons [6,14,15,16,17]. For example, ref. [6] examined whether managerial responses to employee proactivity depend on the type of behavior, that is, whether employees’ suggestions are met with a combative or supportive attitude. However, a few empirical studies examined the managerial side (“supervisor”) only, despite the non-managerial side (“colleagues”) being equally important.

We focus on workplace ostracism because of the unique characteristics of PCWs. They belong to a minority group in number and can be easily alienated due to conflicts with existing permanent employees. In terms of employment status, PCWs are also permanent employees and thus hold a formal status equal to existing permanent employees. However, their expertise and knowledge differ from those of existing permanent employees, and they are contenders who cannot be easily ignored. Therefore, unlike other types of industry-ready workers outsourced from the external labor market, PCWs are seldom blatantly persecuted or harassed by existing permanent employees. However, they are likely to be subjected to the “silent treatment”, rather than blatant harassment. Workplace ostracism could reflect the nature of the potential conflict between PCWs and existing permanent employees accurately.

It has been pointed out that workplace ostracism has a positive relationship with turnover intention [18,19,20]. This study examines whether the conflicts between PCWs and existing permanent employees are the cause of their early resignations.

To that end, we examine the mediating effect of workplace ostracism on the relationship between proactive behavior and turnover intention in the context of portfolio career employment. Additionally, the conditions under which the relationship between proactive behavior and workplace ostracism and between workplace ostracism and turnover intention become noticeable are determined in the boundary condition. 

## 2. Hypotheses Development

### 2.1. Workplace Ostracism

Workplace ostracism refers to “the extent to which an individual perceives that he or she is ignored or excluded by others at work” [18] as well as “individual or a group neglecting to take actions that engage another organizational member when it would be customary or appropriate to do so” [21]. Ref. [18] pointed out that these two different definitions reflect that ostracism is not only defined as “action” or “inaction” but also as “perception”. The former definition focuses on the perpetrators (objective ostracism), and the latter focuses on the victims (subjective ostracism). This article uses the latter because it reflects a wider range of actual ostracism.

As an objective fact, ostracism is divided into three categories based on the relationship between potential “perpetrators” (in this case, existing permanent employees) and potential “victims” (in this case, PCWs). First, PCWs experience ostracism due to intentional “actions” by the existing permanent employees; this is intentional (or purposeful) ostracism. Second, PCWs “perceive” ostracism even when an existing permanent employee does not intend it; this is unintentional (or non-purposeful) ostracism. Third, PCWs do “not perceive” any kind of ostracism even when existing permanent employees commit intentional ostracism. The definition of [21] does not reflect the second case. It also does not distinguish between the first and second cases because the definition regards ostracism as an intentional “action”. On the other hand, the definition of [18], which focuses on the “perception” of the potential “victims”, reflects all three cases. 

The concepts of workplace victimization [22], workplace bullying [23], mobbing at work [24], and workplace harassment [25] are similar to ostracism. However, workplace ostracism has several distinct characteristics.

First, ostracism felt by PCWs (victims) does not necessarily imply the objective existence of intentional “action” by existing permanent employees (perpetrators). Non-purposeful ostracism may occur where interactions between employees are infrequent for reasons such as stress due to work pressures or physical distance between employees [26]. Employee diversity and heterogeneity are also antecedent factors of non-purposeful ostracism [26,27]. PCWs may be aware that existing permanent employees are wary of them. On the other hand, existing permanent employees may lack an awareness of the newcomers’ efforts to interact with them proactively [1]. This is a typical example of non-purposeful ostracism.

Second, even when ostracism is caused by the intentional actions of perpetrators, these tend to be subtle actions [18,26,27,28], such as greetings going unanswered at work [18], which prevent the victims from being a member of the group. Therefore, unlike aggressive behavior, such as ranting and assaults often observed in cases of workplace harassment, bullying, and victimization, ostracism is characterized by the “silent treatment”, which is difficult for the victims to prove [21]. Such “silent treatment” tends to occur more frequently in horizontal relationships, which lack a formal authority and power that can directly control offensive behavior [26,29]. Therefore, unlike verbal abuse or assault, which occurs more frequently in leader–follower relationships that involve power dynamics (e.g., the abusive supervision), the “silent treatment” is primarily characterized by a horizontal relationship among colleagues whose social hierarchy are not clear [29].

Third, workplace ostracism is a means of resolving conflict by controlling and changing the behavior of the victims [26]. The study of concepts similar to workplace ostracism has focused on the organizational flaws that induce this kind of out-grouping. For example, singling out people as examples at the expense of other members, personnel policies that induce fierce competition and stress, the characteristics of perpetrators (such as domineering bosses and mentally abusive colleagues), and the characteristics of victims susceptible to bullying have been identified as antecedent factors of harassment in the workplace [25]. However, workplace ostracism occurs not only for psychological purposes, such as to display aversion toward the victims [30], but also for pragmatic purposes, such as to apply sanctions on and remedies for the attitude and behavior of the victims [31]. According to [26], workplace ostracism can arise from the motive to protect a group by blocking information, resources, and support to impede the target’s “norm-deviant behavior”. From this point of view, ostracism serves as a message that the “norm-deviant behavior” must be corrected to a conforming behavior toward the existing consensus and agreement.

Thus, we can conjecture that workplace ostracism may occur when PCWs display change-oriented behavior that deviates from the existing group norms. For example, adjustment within the workplace can be disrupted when PCWs criticize existing processes and practices in a new organization based on their experience in a previous organization [32]. This may be perceived as a lack of respect for the group norms, implicit knowledge, and practices shared among existing permanent employees. 

### 2.2. Proactive Behavior and Workplace Ostracism

Based on the expertise and experience of industry-ready PCWs, companies expect them to proactively propose new ideas to be put into practice [3]. Ref. [10] defines proactive behavior as “taking initiative in improving current circumstances or creating new ones; it involves challenging the status quo rather than passively adapting to present conditions”. Ref. [10] emphasizes that proactive behavior is applied to both in-role and extra-role behavior. For example, when PCWs working in the sales department voluntarily update the market research materials, contact external expert groups, or grasp new industrial trends, these are examples of in-role proactive behavior. On the other hand, when PCWs focus on the needs of the new market and suggest the expansion of the product lineup, or propose to the developmental department the release of new products or services, these are examples of extra-role proactive behavior.

Ref. [33] categorized two kinds of proactive behavior into in-role proactive behavior (“continuous improvement”) and extra-role proactive behavior (“voice behavior”) based on [10]. In-role proactive behavior is a supportive form of proactive behavior that involves change-oriented actions aimed at improving productivity and functionality based upon the formal performance evaluation criteria [33]. On the other hand, extra-role proactive behavior is a challenging form of proactive behavior that involves constructive change-oriented communication intended to innovative, behavior considered norm-deviant because it challenges the status quo [33]. In contrast to in-role proactive behavior, which is proactive behavior that respects existing norms, extra-role proactive behavior has attributes of norm-deviant behavior in that it raises doubts about the status quo.

We predict that the in-role proactive behavior (“continuous improvement”, [33]) of PCWs causes a positive response from existing permanent employees, while extra-role proactive behavior (“voice behavior”, [33]) causes a negative response, for three reasons.

First, extra-role proactive behavior forms a negative impression, but in-role proactive behavior forms a positive one. Ref. [34] argue that pointing out problems in existing practices may leave a negative impression of a supervisor or colleagues. Ref. [35], who conceptualized voice behavior, emphasize that simply complaining without concrete countermeasures and solutions must be distinguished from voice behavior that promotes constructive change in the organization. However, voice behavior may be perceived as resistance to or complaints about the organizational policy, since it starts with fundamental reflections on and criticisms of the status quo of the organization. Voice behavior can also exacerbate interpersonal relations with management. Ref. [18] reconceptualized voice behavior as a “challenging voice” and “supportive voice”, and he demonstrated that a “challenging voice” has a negative effect on personnel evaluations. On the other hand, in-role proactive behavior that expresses support for the status quo of the organization will create a positive impression because in-role behavior demonstrates a faithful attitude. Related to this, ref. [18] demonstrated that a “supportive voice” has a positive effect on personnel evaluations. 

Second, extra-role proactive behavior increases the work burden of other employees, while in-role proactive behavior reduces it. In-role proactive behavior is limited to the scope of the individual job descriptions, and it does not require the participation of other employees. On the other hand, extra-role proactive behavior requires the participation of other employees because it proposes organizational or group-level change. For example, the proposal to completely alter the task flow of a department would affect not only the voicer, but other employees as well. Additionally, cooperating with extra-role proactive behavior implies organizational citizenship behavior (OCB) among other employees. Moreover, OCB, as extra-role behavior, adversely affects role overload, job stress, and the work–life balance [14,36]. The context of OCB is crucial, because it helps explain why existing permanent employees cannot explicitly oppose the newcomer’s extra-role proactive behavior, since OCB tends to be accepted as a virtue. Therefore, it is not possible to openly oppose it, even if one is annoyed with the extra-role proactive behavior. This can lead to the silent treatment. Moreover, the challenging attributes of extra-role proactive behavior may interfere with the execution of other members’ tasks. Ref. [8] demonstrated that innovative behavior has a positive effect on interpersonal conflicts with colleagues. On the other hand, in-role proactive behavior, which pursues the improvement of efficiency, reduces the workload of other employees. For example, if in-role proactive behavior optimizes a part of the task flow responsible for a bottleneck affecting overall efficiency, the work of other employees may be improved.

Third, extra-role proactive behavior leads to win–lose relationships in response to conflicts of interest with colleagues, whereas in-role proactive behavior forms win–win relationships. Extra-role proactive behavior tends to facilitate access to the limited resources (e.g., budget, personnel, time) of their organization [37]. Extra-role proactive behavior also makes it possible for the voicer to secure a high status and a reputation as an innovator [38,39,40,41,42]. In general, offering innovative ideas serves as a competitive advantage that differentiates the voicers from other employees, thereby exerting a positive effect on promotion, job assignments, social status, and performance evaluations [10,11,12,35,38,39]. 

The problem is that obtaining resources via extra-role proactive behavior puts employees in a competitive relationship with others. When PCWs secure more resources, this may reduce the access to resources of other permanent employees, because resources are limited [37]. Moreover, PCWs who secure status and a reputation as innovators within the company due to their extra-role proactive behavior may gain an advantageous position regarding internal promotion. Competition and conflict between PCWs and existing permanent employees [3] that may arise due to concerns that PCWs will deprive existing permanent employees of the achievements and status they have accumulated over the years [4] have been cited as problems accompanying portfolio career recruitment. On the other hand, if productivity is improved through in-role proactive behavior, the benefits are not enjoyed only by PCWs, but are shared with existing permanent employees. 

The above evidence suggests that existing permanent employees may react in a direction contrary to that intended by the proactive behavior of PCWs. However, even if existing permanent employees are dissatisfied with the extra-role proactive behavior of PCWs, this will not automatically lead to workplace ostracism, because perpetrating it can incur the following variety of potential “costs” [26,29]. First, workplace ostracism can cause emotional distress such as discomfort, guilt, and stress to perpetrators for their “unethical behavior” [26,29,43]. Second, workplace ostracism can be subject to disciplinary action and punishment if managers or the personnel department discover it, since it can hinder organizational effectiveness [44,45,46]. Therefore, workplace ostracism requires that perpetrators have a clear reason to engage in it, as they need to convince themselves that their unethical and counterproductive behavior is justified [26,30]. This is explained in relation to the three motives for workplace ostracism below.

Firstly, workplace ostracism can be “justified” by perpetrators themselves for the sake of stability, harmony, or efficiency when a group is challenging norm-violators who are ignoring implicit rules [26,30,31]. Generally, norm-deviant behavior tends to be seen as something that inhibits the efficiency of the group [47]. Workplace ostracism can informally transmit the “signal” that existing permanent employees can no longer overlook the tension and confusion caused by the deviant behavior of PCWs [26,30]. 

Any workplace ostracism occurring from such motives is a kind of unethical pro-organizational behavior [48,49] that is contrary to the social norms, customs, ethics, and laws that ensure the functioning of the organization. This description might seem contradictory in that extra-role proactive behavior (“voice behavior”) is generated out of a prosocial motive of contributing to the performance of the organization and group. However, prosocial motives have attributes that cannot be observed from the outside, and extra-role behavior can be perceived by other employees as personal ranting behavior, finding fault with everything, and not cooperating with matters on which everyone agrees. Hence, opposition to extra-role behavior can also stem from a prosocial motive [50]. For example, ref. [30] demonstrated that workplace ostracism has a positive relationship with unethical behavior in the workplace. Further, when unethical pro-organizational behavior is seen primarily in employees with strong organizational identification [49,51] or with a strong need for social inclusion [52], the workplace ostracism can be considered to express a sense of mission toward the organization. Moreover, the perpetrators of such ostracism will likely be open to silent treatment against the norm violators because they may not recognize the guilt associated with such unethical behavior.

Next, workplace ostracism occurs when other employees perceive relationship conflicts with the norm violators [30]. The confusion and tension caused by deviant behavior cause a negative response to the norm violators. Amid such tense interpersonal relationships, workplace ostracism will occur when no countermeasures are available other than changing the deviant behavior [26]. Resistance to extra-role proactive behavior that encourages constructive changes cannot be made officially such as in a business meeting or workplace gathering. It is also difficult for PCWs to communicate dissenting views through informal communication, because they are not closely connected to existing permanent employees. If there is no other way to resolve the relationship conflict, existing permanent employees will rely on the simple “cognitive solution” of trying to minimize the frequency of contact with the “troublemakers” and ignoring the source of the conflict [26,30,31].

Moreover, workplace ostracism arising from a wish to avoid relationship conflicts is more of a defensive attempt to protect oneself from turmoil and tension caused by deviant behavior than an aggressive act against norm violators. Particularly, workplace ostracism arising from defensive motives often occurs in organizations with conflict-avoidant cultures, climates, or policies, and in members who try to avoid conflict as much as possible [26]. This kind of workplace ostracism is expected to appear as a form of “sideliner” behavior designed to silence or neutralize the intentional workplace ostracism of other employees. Alternatively, this type of ostracism may have unintended ostracism attributes, as it is not an aggressive attempt to exclude certain members but to foster psychological well-being. In any case, such perpetrators are considered to be the most moderate in terms of the intensity, frequency, and effect of workplace ostracism. 

Finally, workplace ostracism also has self-interested motives based on the potential personal gain enabled by the behavior, and it also occurs when employees share this personal gain. Thus, workplace ostracism that blocks information, resources, and support for the voicer [26] reduces the work burden, job stress, and negative impacts on the work–life balance [36] arising from the OCB of cooperating with extra-role proactive behavior. Competitors within the company can also prevent PCWs from gaining promotions, job assignments, and performance evaluation benefits through their extra-role proactive behavior.

Moreover, workplace ostracism due to motives based on personal gain appears as opportunistic behavior that passively stimulates the silent treatment. Here, the opportunistic behavior comprises participation in workplace ostracism when the personal “gain” it produces exceeds the “cost”. Employees will reluctantly participate in the silent treatment to the extent that the risk associated with workplace ostracism (e.g., the possibility of in-house disciplinary action and punishment) can be minimized. 

The above explanation suggests that workplace ostracism occurs due to various motives. Moreover, the fact that several motives can be combined strengthens the relationship between workplace ostracism and proactive behavior, as workplace ostracism can be experienced via the silent treatment imposed in various forms, degrees, and intensity [26]. Therefore, we present the following hypotheses:

**Hypothesis** **1a.** *The extra-role proactive behavior of PCWs has a positive effect on their perceived workplace ostracism.*

**Hypothesis** **1b.** *The in-role proactive behavior of PCWs has a negative effect on their perceived workplace ostracism.*

### 2.3. Moderated Effect of Employee–Organization Exchange Relationship on Relationship between Workplace Ostracism and Proactive Behavior

Here, the employee–organization exchange relationship is presented as a boundary condition for moderating the mechanism of WO. We focus on the exchange relationship between the two due to the three WO motives presented in Hypothesis 1. The employee–organization exchange relationships are divided into two types. The first is an economic exchange relationship, which involves only limited cooperation from employees as clearly stated in advance in the employment contract and motivated by a narrow, short-term incentive system. The second is a social exchange relationship, which involves the voluntary cooperation of employees on a comprehensive level that is not clearly defined in the employment contract and is motivated by a broad, long-term incentive system. Ever since full-scale PCW employment began in the 1990s, Korean companies’ personnel systems have been changing from a social exchange relationships basis (i.e., a system of lifetime employment and job security) to one of economic exchange relationships (i.e., flexible and performance-based practices; [53]. Below, we explain why such changes at an institutional level are moderated by the relationship between proactive behavior and workplace ostracism.

First, the mutual reciprocity norm emphasized in the social exchange relationship strengthens ostracism motives. As mentioned, workplace ostracism has attributes of unethical pro-organizational behavior. Ref. [49] demonstrated a positive relationship between organizational identification and unethical pro-organizational behavior, described as the “dark side” of organizational identification. The existing permanent employees with strong organizational identification may even perceive unethical pro-organizational behavior as a special form of gratitude that only “selected” employees can offer. For this reason, workplace ostracism against norm violators can be seen as a great opportunity to show gratitude to the organization through good intentions, and it will strengthen the motivation to protect its norms.

Second, the task interdependence emphasized in the social exchange relationship strengthens the sideliner workplace ostracism motive. Here, “interdependence” refers to a structure in which the desires, thoughts, attitudes, and behaviors of one employee affect all other employees [54]. In the social exchange relationship, there is a tendency to adopt the team structure as the basic unit of intra-organizational communication (e.g., self-managed teams, small group activities, roundtable discussions in departments). The social exchange relationship facilitates an ambiguous scope of work, flexible job rotation, and multi-skilling, and a cooperative framework that supports other employees is developed as necessary. For this reason, it is predicted that the degree to which norms and roles are shared by employees is higher when the social exchange relationships are formed. When these relationships are formed, the conflict arising from the uncertainty caused by the innovative behavior described by [8], problems with the cooperative framework, and the time and effort required to avoid an escalation of commitment will also increase. These factors will strengthen the sideliner ostracism motivation to avoid relationship conflicts caused by “troublemakers”.

Third, the team-based structure emphasized in social exchange relationships strengthens the opportunist ostracism motive. Companies that adopt the social exchange relationship as a fundamental principle of their personnel system tend to emphasize employee contributions at the group level rather than at the individual level [55,56]. In the social exchange relationship, task setting, performance evaluations, and reward systems tend to be determined according to the contribution made by each department rather than by individual job performance [2,37,56]. Therefore, in a social exchange relationship, voicers can gain easy access to resources, because limited resources can be concentrated through the realization of extra-role proactive behavior at the discretion of the department. The contribution to their department made by extra-role proactive behavior will be appraised highly during the year-end performance evaluation. In general, performance evaluations consist of relative measures. All these factors will increase the possibility of a conflict of interest due to extra-role proactive behavior and strengthen the opportunist ostracism motive. 

Therefore, we present the following hypotheses:

**Hypothesis** **2a.** *The positive relationship between the extra-role proactive behavior of PCWs and their perceived workplace ostracism is stronger in a social exchange relationship than it is in an economic exchange relationship.*

**Hypothesis** **2b.** *The negative relationship between the in-role proactive behavior of PCWs and their perceived workplace ostracism is stronger in a social exchange relationship than it is in an economic exchange relationship.*

### 2.4. Workplace Ostracism and Turnover Intention

Here, we propose that workplace ostracism has a positive effect on the turnover intention of PCWs. As mentioned, the positive relationship between workplace ostracism and turnover intention has been demonstrated [18,19,20]. This relationship occurs for two reasons.

First, from the viewpoint of social identity theory, workplace ostracism reduces the sense of affiliation, respect, sense of control, and meaningful existence that are vitally important for organizational life. Workplace ostracism informally invalidates the authority, autonomy, and responsibility the target should receive from the organization, preventing the target from forming a psychological attachment to the organization or group [29] and making it impossible to acquire a meaningful presence through work [57].

Second, according to job stress theory, workplace ostracism causes psychological distress through the severance of social relations, leading to various psychological responses such as anger, emotional scars, frustration, loss of self-esteem, confusion, and anxiety [18,21,58,59,60]. We thus propose the following:

**Hypothesis** **3.** *Perceived workplace ostracism exerts a positive effect on the turnover intention of PCWs.*

### 2.5. Moderated Effect of Newcomers’ Status in a New Organization on the Relationship between Workplace Ostracism and Turnover Intention

The expansion of portfolio career employment has increased the number of PCWs who enjoy high status in a new organization. We focus on the newcomer’s status as a boundary condition moderating workplace ostracism’s impact on turnover intention. Specifically, we propose that the positive relationship between PCWs’ perceived ostracism and their turnover intention becomes stronger as their formal job ranks and informal decision-making influence within the new organization increase. 

We divide status into job rank and job title. Job rank refers to rank order, position, and influence in the organizational hierarchy. Although the term used to indicate the designation differs depending on the company, job ranks such as “general manager” can be considered useful proxy variables indicating the employee’s formal rank. For example, job titles such as “section chief or equivalent”, “deputy general manager or equivalent”, and “general manager or equivalent” can be considered proxy variables indicating the employee’s job rank. Job title refers to the range of roles, responsibilities, and authorities within the organization. For example, job titles such as “head of sales team 1”, “head of sales team 2”, and “head of sales team 3” are examples of the middle manager class of the sales department. However, the three middle manager positions will have different levels of decision-making influence depending on their roles, their importance, and the responsibility of their job description. 

The validity of job rank and title as proxy variables reflecting hierarchical status in a new organization will vary according to the form of the organizational structure. For example, in companies with a hierarchical organizational structure (traditional Korean HR practices) with well-defined and sub-divided job ranks and command and control systems, job ranks may be more important than job titles. On the other hand, companies with a flat organizational structure (contemporary Korean HR practices) emphasize expertise and discretion, and hence, job titles may be more important than job ranks. 

We use the formal job rank and informal decision-making influence as proxy variables corresponding to the job rank and job title. Here, decision-making influence refers to the “influence that an employee has over decisions that affect him/her or the work that s/he does”.

We propose that the positive effect of workplace ostracism on turnover intention is influenced by differences across perceived organizational structure.

First, workplace ostracism becomes more likely to be discovered by other employees whose victims occupy a higher organizational position. The higher the status of victims, the higher the visibility in the company [61]. High-status members are located in the center of the communication network, meaning that their decision-making has a strong influence on other employees. Consequently, other employees will be particularly interested in the decisions high-status members make, as well as their context, motivation, and purpose. Such conditions will amplify the shame, frustration, and emotional exhaustion caused by workplace ostracism. Especially, Korea’s socio-cultural environment has a relatively high level of power distance and collectivism. Thus, high-status employees require dignity, prestige, and faith. Therefore, the abovementioned effects described via the social identity argument and job stress theory will increase for such employees (victims).

Second, the higher the victim’s status, the more likely it is that workplace ostracism will hinder job performance. The higher the status of the member, the higher the interdependence of the tasks and the more ambiguous the boundaries of the responsibilities [62]. Moreover, the higher the status in a new organization, the higher the uncertainty between the means and results of the performance. Coping with such uncertainty requires obtaining information, resources, and support from other employees. This is a catalyst that increases the negative effect that workplace ostracism’s blocking of information, resources, and support exerts on job performance. Therefore, we present the following hypotheses:

**Hypothesis** **4a.** *The positive relationship between the perceived workplace ostracism and turnover intention is strengthened as the newcomer’s formal job rank increases.*

**Hypothesis** **4b.** *The positive relationship between the perceived workplace ostracism and turnover intention is strengthened as the newcomer’s decision-making influence increases.*

**Figure 1:**
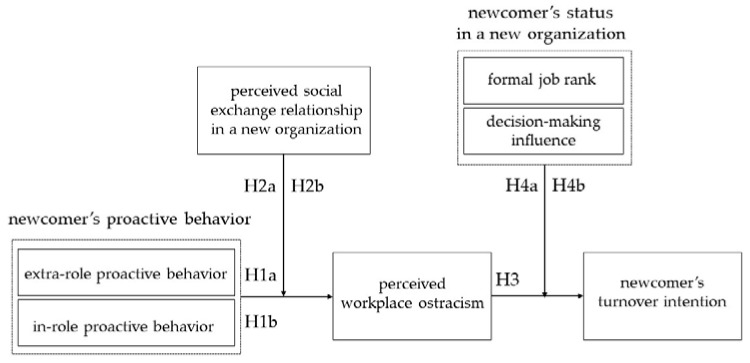
Hypothesized model.

Figure 1 summarizes the relationships proposed in the hypotheses. 

## 3. Method

### 3.1. Research Context: Portfolio Career Employment in South Korea

In the 1990s, the personnel systems of Korean companies changed through the introduction of employment practices that utilize human resources from external labor markets, including PCWs [53,63,64,65,66]. The traditional Korean HR practice was to internalize human resources via a recruitment system centered on new graduates, long-term employment practices, and a seniority-based wage and promotion system. Therefore, the core talent in Korean companies consisted of long-term permanent employees who had joined as new graduates. Those long-term permanent employees shared values such as a sense of loyalty to the company, collective solidarity based on year of entering the company, and respect for the power distance formed on the basis of a seniority system. However, employment adjustment, performance-based wage and promotion systems, and employment practices utilizing the external labor market were introduced, triggered by the economic crisis of 1997, and organizational order began to disintegrate. In particular, personnel policies emphasizing a performance-based wage system (expertise) were introduced to replace the seniority-based wage system (tenure), and it became common for PCWs to hold core positions. 

The case of Korean PCWs is an appropriate sample with which to verify the research model shown in Figure 1. While PCWs are numerically a minority group, they became fierce competitors in the race for promotions, as their employment status is equal to that of existing permanent employees. These two types of permanent employees represent fundamentally incompatible HR logics, and their collective status may change depending on the logic applied. Conflicts of interest due to the behavior of PCWs, relationship conflicts, and the collapse of shared norms may not be caused only by individual behaviors but also by the pressure exerted by the changes due to the portfolio career employment system.

### 3.2. Samples and Procedure

An online survey was implemented in 2015 to collect data from Korean PCWs who registered in a professional monitoring pool. Using a screening survey, we operationally defined PCWs based on the following eight conditions. First, his/her employment status is that of a permanent employee. Second, the size of his/her current company is 100 employees or more. Third, hiring is conducted through the headhunting or recruitment of PCWs only. Fourth, his/her tenure in the current company is between six months and five years. Fifth, his/her tenure in the previous company was between one and seven years. Sixth, his/her formal job rank in the current company is “senior staff/assistant manager or equivalent”, “supervisor/manager or equivalent”, or “assistant general manager or equivalent”. Seventh, his/her formal job rank immediately before the most recent job change was not a “junior staff or equivalent” but that of “general manager or the equivalent” or higher. Eighth, his/her age is no higher than 60 years. The main section of the survey contained questions regarding demographic and career information and the main variables shown in Figure 1. The survey screening continued until the number of respondents meeting the above conditions reached 300. A total of 265 questionnaires were collected, and four provided unusable answers, resulting in a final sample of 261 (for an 87% response rate). 

### 3.3. Measures

For all research variables, the participants self-reported the survey questions on a Likert-type scale ranging from “strongly disagree” (1) to “strongly agree” (7). As all participants were Korean, we translated the survey items. To reduce cultural bias and error in the translation processes, we used the iterative back-translation method [67]. 

#### 3.3.1. Proactive Behavior

Extra-role proactive behavior was assessed using [35] six-item scale. Sample items include “I communicate my opinion about work issues to others in this group even if my opinion is different and others in the group disagree with me”. Among these six items, we excluded one item (“I develop and make recommendations concerning issues that affect this work group”; See Appendix A for detailed item scales), since the results of confirmatory factor analysis were poor due to cross-loading with extra-role proactive behavior and in-role proactive behavior. The Cronbach’s alpha for this scale was 0.91. In-role proactive behavior was assessed employing [33] four-item scale based on [10] conceptualization of in-role proactive behavior. Sample items include “I devise ways to improve processes” (See Appendix A for detailed item scales). The Cronbach’s alpha for this scale was 0.89.

#### 3.3.2. Workplace Ostracism

Workplace ostracism was assessed adopting [18] 13-item scale. Sample items include “others left the area when I entered”. Among these 13 items, we excluded seven because some reflected the cultural context (“others at work did not invite you or ask you if you wanted anything when they went out for a coffee break”; See Appendix A for detailed item scales). The Cronbach’s alpha for this scale was 0.95.

#### 3.3.3. Turnover Intention

Turnover intention was assessed using [68] five-item scale. Sample items include “I intend to leave this organization soon”. (See Appendix A for detailed item scales). The Cronbach’s alpha for this scale was 0.93.

#### 3.3.4. Perceived Social Exchange Relationship in a New Organization

The employee–organization relationship in HR practices was estimated using [56] seven-item scale. Sample items include “This organization evaluates employees based on unit performance” (see Appendix A for detailed item scales). The Cronbach’s alpha for this scale was 0.95. Using the k-means clustering method, we categorized the exchange relationship into two kinds: the economic exchange relationship and the social exchange relationship. Among the 261 participants, 153 (58.62%) had worked in a social exchange relationship and 108 (41.38%) had worked in an economic exchange relationship.

#### 3.3.5. Newcomer’s Status in a New Organization

Formal job rank was estimated based on the participants’ self-reporting. Companies use different job rank designations due to Korean companies’ recent “simplification of job rank”. Therefore, respondents were asked to choose the closest job rank based on the extent of their duties and degree of responsibility. Formal job ranks were divided into three categories: “senior staff/assistant manager or equivalent”, “supervisor/manager or equivalent”, and “deputy general manager or equivalent”. Koreans customarily call these job ranks “JooIm/DaeRi”, “KwaJang”, and “ChaJang” respectively. Even companies that have “simplified job rank” maintain the conventional designations externally because information about job rank allows Koreans to understand their formal status in a new organization. Decision-making influence was measured using [69] three-item scale, with one new item added. Sample items include “I have influence over decisions in this organization about ways to improve quality”. The additional item was “Overall, I have influence over decision-making processes in this organization”. (See Appendix A for detailed item scales). The Cronbach’s alpha for this scale was 0.91.

#### 3.3.6. Control Variables

As a previous study has shown that gender, age, tenure in the current organization, education level, firm size (number of employees), salary level in the current organization, industry, and occupation can influence PCWs’ proactive behavior and their interactions with coworkers and supervisors [2,37], we controlled for all of these variables. Gender, industry, and occupation were dummy coded. Salary level, measured in Korean won, was log-transformed to fit with a normal distribution. 

### 3.4. Data Analysis

We followed previous studies in our approach to the analysis [39,70]. First, we conducted confirmatory factor analysis to assess the measurement properties of the main variables shown in Figure 1. The goodness of fit was assessed comprehensively using four indexes. The value for badness of fit (χ^2^/df), the ratio of the chi-square (χ^2^) divided by the degrees of freedom (df), should be smaller than 5.0 [71]. The comparative fit index (CFI) should be greater than 0.90, and a greater value indicates a better fit [72]. The values for the standardized root mean residual (SRMR) and the root mean square error approximation (RMSEA) should be smaller than 0.1 and 0.08, respectively, and smaller values indicate a better fit [72,73]. 

Hypotheses 1 and 3 were tested using multiple regression analyses. Additionally, we adopted bootstrap-based mediation analysis to confirm the significance of indirect effects [74]. We tested hypotheses 2 and 4 using a bootstrap-based conditional process model [75] to estimate a two-stage moderated mediation model. This regression approach based on bootstrapping sampling is more reliable in detecting the statistical significance of conditional indirect effects [76]. 

## 4. Results

We tested our hypothesized seven-factor model against other alternative models. Table 1 shows the CFA results of the hypothesized model. Overall, our hypothesized model (Model 1) showed satisfactory goodness of fit (χ^2^/df = 2.122, SRMR = 0.045, RMSEA = 0.066, CFI = 0.940) and a better fit than in the alternative models. The factor model used in this study was compared with alternative factor models and was found to have higher overall validity (*p* < 0.001). Lastly, Harman’s single factor test [77] was conducted to check for the existence of common method variance. We conducted exploratory factor analysis (EFA) to confirm whether a general factor explained most (50%) of the total variance. We found that a single factor explained only 34.45% of the total variance. Based on these results, the seven-factor model shown in Figure 1 was deemed to have no serious validity issues. 

Means, standard deviations, and correlational coefficients for all variables are presented in Table 2. Our two types of proactive behavior were highly correlated (*r* = 0.84, *p* < 0.001). A recent study [39] that classified voice behavior into two types—supportive and challenging—revealed a high correlation (r = 0.75, *p* < 0.01) between them and analyzed them the same way we do. In other words, a high correlation between variables does not necessarily hinder the divergent validity of the study. However, we confirmed that there were no serious multicollinearity issues via a post-estimation test using the variance inflation factor. 

The results of the multiple regression analysis are presented in Table 3. As shown, extra-role proactive behavior was positively and significantly related to workplace ostracism (*B* = 0.457, *p* < 0.001), supporting Hypothesis 1a, and in-role proactive behavior was negatively and significantly related to workplace ostracism (*B* = −0.649, *p* < 0.001), supporting Hypothesis 1b. 

Hypothesis 2 expected that the employee–organization relationship in HR practices moderates the relationship between PCWs’ proactive behavior and their perceived workplace ostracism. Extra-role proactive behavior and in-role proactive behavior were mean-centered before input into the equation and computation of the interaction term. The results of the moderated regression analyses (see Table 3) show that the interaction term between extra-role proactive behavior and the social exchange dummy was positively and significantly related to workplace ostracism (*B* = 0.250, *p* < 0.05), supporting Hypothesis 2a. The analyses of the conditional effect of extra-role proactive behavior on workplace ostracism showed that it was stronger for the social exchange (effect = 0.998, t = 4.836, *p* < 0.001) than for the economic exchange (effect = 0.110, t = 0.437, non-significant), indicating that extra-role proactive behavior is riskier in a social exchange relationship than in an economic exchange relationship.

**Figure 2:**
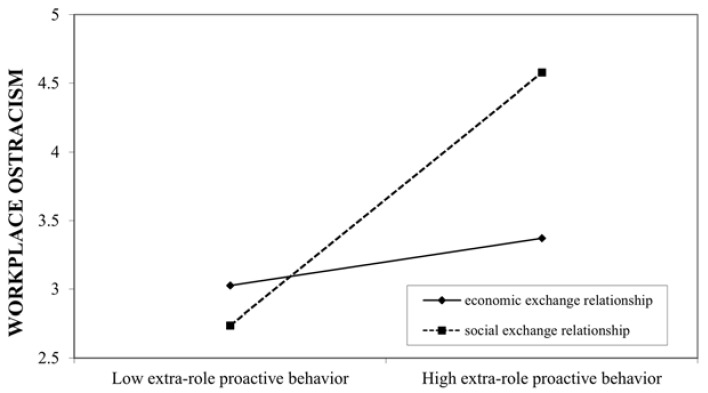
The interactive effect of extra-role proactive behavior and exchange relationship in HR practices on workplace ostracism.

**Figure 3:**
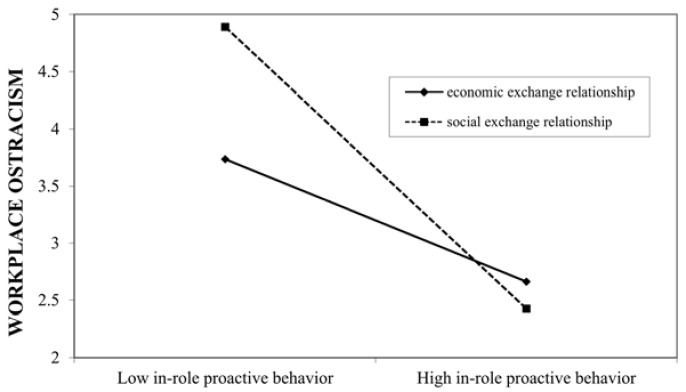
The interactive effect of in-role proactive behavior and exchange relationship in HR practices on workplace ostracism.

The interaction term between in-role proactive behavior and the social exchange dummy was negatively and significantly related to workplace ostracism (*B* = −0.236, *p* < 0.05), supporting Hypothesis 2b. The conditional effect of in-role proactive behavior on workplace ostracism was stronger for the social exchange (effect = −1.376, t = −6.468, *p* < 0.001) than for the economic exchange (effect = −0.519, t = −2.081, *p* < 0.05), indicating that in-role proactive behavior is more beneficial in the social exchange relationship than in the economic exchange relationship. We plotted these relationships in Hypothesis 2 (see Figure 2 and Figure 3).

Regarding Hypothesis 3, workplace ostracism was positively and significantly related to turnover intention (*B* = 0.645, *p* < 0.001); thus, Hypothesis 3 is supported. 

Hypothesis 4 expected that the newcomer’s status in a new organization moderates the positive relationship between workplace ostracism and turnover intention. Workplace ostracism and decision-making influence were mean-centered before input into the equation and computation of the interaction term. The results of the moderated regression analyses (see Table 3) show that the interaction term between workplace ostracism and “senior staff/assistant manager or equivalent” on turnover intention was non-significant (*B* = 0.006, non-significant) and that the interaction term between workplace ostracism and “deputy general manager or equivalent” on turnover intention was positively but only marginally significantly related to turnover intention (*B* = 0.084, *p* < 0.1), partially supporting Hypothesis 4a. The conditional effect of workplace ostracism on turnover intention was stronger for “deputy general manager or equivalent” (effect = 0.725, t = 7.708, *p* < 0.001) than for “supervisor/manager or equivalent” (effect = 0.572, t = 7.766, *p* < 0.001) or “senior staff/assistant manager or equivalent” (effect = 0.476, t = 4.778, *p* < 0.001). However, the difference between “supervisor/manager or equivalent” and “senior staff/assistant manager or equivalent” was not statistically significant. 

**Figure 4:**
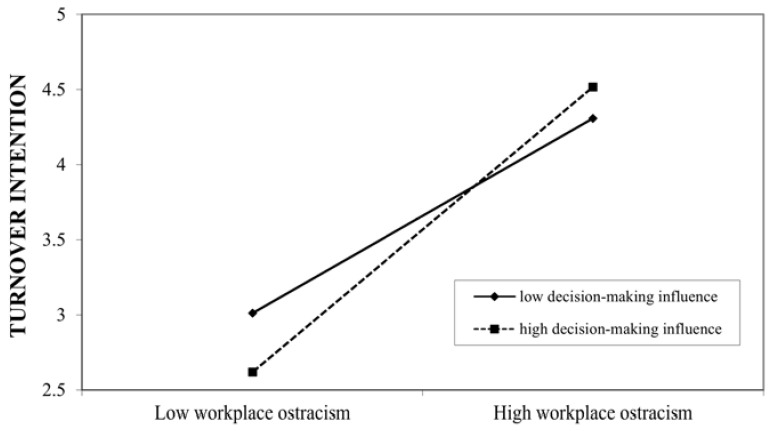
The interactive effect of workplace ostracism and decision-making influence on turnover intention.

The interaction term between workplace ostracism and decision-making influence was positively and significantly related to turnover intention (*B* = 0.129, *p* < 0.05), supporting Hypothesis 4b. The conditional effect of workplace ostracism on turnover intention was stronger at high levels of decision-making influence (effect = 0.682, t = 11.116, *p* < 0.001) than at low levels of decision-making influence (effect = 0.465, t = 6.249, *p* < 0.001), indicating that workplace ostracism is more harmful in higher informal status than in lower informal status. We plotted these relationships in Hypothesis 4b (see Figure 4).

To test the moderated mediation model shown in Figure 1 as a supplement analysis, we used a bootstrap mediation method with 5000 samples with replacement and percentile bootstrap confidence intervals. The results for the indirect, direct, and total effects of proactive behavior on turnover intention through workplace ostracism are presented in Table 4.

The indirect effect of extra-role proactive behavior on turnover intention through workplace ostracism was significant and positive (effect = 0.439; 95% confidence interval (0.236, 0.648)), as indicated by the confidence interval excluding zero. The indirect effect of in-role proactive behavior on turnover intention through workplace ostracism was significant and negative (effect = −0.607; 95% confidence interval (−0.840, −0.394)). These results show that workplace ostracism mediates the two types of proactive behavior and turnover intention.

The total effect of extra-role proactive behavior on turnover intention through all potential mediators, including workplace ostracism, was not significant (effect = 0.242; 95% confidence interval (−0.079, 0.562)). As mentioned, extra-role proactive behavior has both positive and negative effects on outcome variables. This result reflects all potential paths, though this study focuses on the partially negative path measured by workplace ostracism. According to the conventional mediation model of [9], the significance of the total effect between the independent variable and the dependent variable is a prerequisite for a mediation test. However, recent research has pointed out that there are sometimes many indirect pathways and the total effect is not always significant when the signs of the pathways differ [77,78,79]. For example, it can be predicted that many indirect pathways have a positive sign and that a large number of indirect pathways have a negative sign. Thus, if an offset effect can be predicted among the indirect effects, the total effect may not be significant. Therefore, the fact that the total effect is not significant does not mean that there is no mediation effect. On the other hand, the effect of in-role proactive behavior on turnover intention through all potential mediators, including workplace ostracism, was significant and negative (effect = −0.518; 95% confidence interval [−0.852, −0.183]). 

Table 4 shows that the indirect effect of extra-role proactive behavior on turnover intention via workplace ostracism was significant for the social exchange relationship (effect = 0.595; 95% confidence interval (0.338, 0.883)) but not for the economic exchange relationship (effect = 0.066; 95% confidence interval (−0.281, 0.378)). Following [80], we further tested the significance of the difference between these two indirect effects and found that it was significant (difference = 0.529, 95% confidence interval (0.146, 0.883)). Table 4 also shows that the indirect effect of in-role proactive behavior on turnover intention via workplace ostracism was significant for the social exchange relationship (effect = −0.832; 95% confidence interval (−1.099, −0.584)) but not for the economic exchange relationship (effect = −0.314; 95% confidence interval (−0.634, 0.016)). A significance test for the difference between these two indirect effects found that it was significant (difference = −0.518, 95% confidence interval (−0.926, −0.156)). 

Table 4 shows that the indirect effect of extra-role proactive behavior on turnover intention in a two-stage mediation was significant regardless of the respondent’s formal job rank (“senior staff/assistant manager or equivalent”: effect = 0.363, 95% confidence interval (0.202, 0.535); “supervisor/manager or equivalent”: effect = 0.430, 95% confidence interval (0.266, 0.604); “deputy general manager or equivalent”: effect = 0.497, 95% confidence interval (0.305, 0.698)). A significance test for the difference between these three indirect effects found that it was not significant (difference between “senior staff/assistant manager or equivalent” and “supervisor/manager or equivalent” = 0.181, 95% confidence interval (−0.187, 0.243); difference between “senior staff/assistant manager or equivalent” and “deputy general manager or equivalent” = 0.157, 95% confidence interval (−0.040, 0.406)). 

Proactive behavior on turnover intention in a two-stage mediation was significant regardless of the respondent’s formal job rank (“senior staff/assistant manager or equivalent”: effect = −0.535, 95% confidence interval (−0.862, −0.223); “supervisor/manager or equivalent”: effect = −0.561, 95% confidence interval (−0.803, −0.334); “deputy general manager or equivalent”: effect = −0.761, 95% confidence interval (−1.065, −0.477)). A significance test for the difference between these three indirect effects found that it was not significant (difference between “senior staff/assistant manager or equivalent” and “supervisor/manager or equivalent” = −0.026, 95% confidence interval (−0.343, 0.283); difference between “senior staff/assistant manager or equivalent” and “deputy general manager or equivalent” = −0.226, 95% confidence interval (−0.549, 0.050)).

Table 4 also shows that the indirect effect of extra-role proactive behavior on turnover intention in a two-stage mediation was significant for not only a low level of decision-making influence (effect = 340; 95% confidence interval (−0.156, 0.544)) but also a high level (effect = 0.498; 95% confidence interval (0.277, 0.728)). A significance test for the difference between these three indirect effects found that it was significant (difference = 0.078, 95% confidence interval (0.008, 0.169)). Additionally, the indirect effect of in-role proactive behavior on turnover intention in a two-stage mediation was significant for not only a low level of decision-making influence (effect = −0.477; 95% confidence interval (−0.751, 0.243)) but also a high level (effect = −0.698; 95% confidence interval (−0.952, −0.450)). A significance test for the difference between these three indirect effects found that it was significant (difference = −0.113, 95% confidence interval (−0.222, −0.013)).

## 5. Discussion

A few studies on employee proactivity focused on the potential risks of how dangerous it is to speak up to managers [6,15]. However, to the best of our knowledge, no study examined the relationship between proactive behavior and colleague ostracism, especially in the context of newcomers. Our article suggests that not all forms of a newcomer’s proactive behavior receive favorable results. We found that colleagues’ responses to the newcomer’s proactivity depend on the type of the latter’s behavior, exchange relationship in a new organization, and their organizational status.

We analyzed the conflict arising between PCWs and existing permanent employees from the viewpoint of workplace ostracism. We proposed that the extra-role proactive behavior of PCWs, as an antecedent factor of perceived workplace ostracism, exerts a positive effect on workplace ostracism (Hypothesis 1a) and that in-role proactive behavior exerts a negative effect on perceived workplace ostracism (Hypothesis 1b). We also predicted that the positive relationship between extra-role proactive behavior and workplace ostracism is stronger in a social exchange relationship than it is in an economic exchange relationship (Hypothesis 2a) and that the negative relationship between sustained improvement and workplace ostracism is stronger in a social exchange relationship than it is in an economic exchange relationship (Hypothesis 2b). We also proposed that perceived workplace ostracism exerts a positive effect on newcomer’s turnover intention (Hypothesis 3). We also predicted that the positive relationship between workplace ostracism and turnover intention becomes stronger as the status of PCWs increases. Specifically, a job rank showing a high formal status within the company (Hypothesis 4a) and a decision-making influence showing informal status (Hypothesis 4b) were predicted to strengthen the relationship between workplace ostracism and turnover intention. The results of the empirical analysis showed that all hypotheses except Hypothesis 4a were partially supported. These findings, obtained from a two-stage moderated mediation model, offer the contributions described below.

### 5.1. Theoretical Contributions

Our results show that a newcomer’s proactive behavior has contrasting effects on the reactions of existing employees. Few studies have examined whether proactive behavior causes conflict among colleagues, likely because they have implicitly assumed that proactive behavior is useful for both the organization and employees. For example, the central premise of studies related to “voice behavior”, which is a narrower concept than proactive behavior, is that it is useful not only for organizations and departments, but also for the employees who voiced themselves [81]. However, ref. [82] suggested that voicers are regarded as complainers and troublemakers and point out that such employees may face formal sanctions such as negative performance appraisals and job assignments. Ref. [81] points out that the results of research attempting to predict the positive and negative effects of “voice behavior” are mixed. Ref. [6] found that a “supportive voice” has a positive effect and a “challenging voice” has a negative effect on performance appraisals. Thus, although research has been conducted on the formal sanction mechanism related to extra-role proactive behavior, no studies have examined informal sanction mechanisms. Our study demonstrated that informal sanctions (“workplace ostracism”) are reduced in cases where proactive behavior is supportive of existing norms and rules, but norm-deviant behavior will become the target of informal sanctions.

Concerning workplace aggression, this study shows that the motive of workplace ostracism perpetrators is not only an evil intention to malign the victims; they sometimes have “reasonable” grounds. We presented three groups: a group of bellwethers with a sense of mission towards the organization, a self-defensive group that tries to avoid relationship conflicts with troublemakers, and an opportunist group that seeks personal gain through workplace ostracism.

Following previous studies on unethical pro-organizational behavior, we suggest that informal sanctions against extra-role proactive behavior may arise from the prosocial motivation to contribute to the functioning of the organization. This leads to problems for two reasons. First, those leading the workplace ostracism may be absorbed by their cause and may not be aware of the unethical nature of the workplace ostracism. If our study is true, such “self-confidence” may strengthen the sustainability of the workplace ostracism. Second, other members may become attuned to the bellwether. The just cause presented by the bellwether may convince others that workplace ostracism is a “necessary evil” for organization and group functioning. This will allow employees to avoid their moral responsibility for excluding others to avoid conflicts out of embarrassment or self-interest. If this effect continues over the long term, an organizational climate and culture will develop that will restrain changes and innovation within the organization, which may threaten the long-term survival or development of the organization.

This study also clarifies that social exchange relationships do not necessarily lead to positive outcomes. Studies on high-performance work practices discuss the social exchange relationship formed out of trust relationships between organizations and employees as an important managerial resource contributing to organizational performance [83,84,85]. However, ref. [53] warn that the in-group harmony unique to the personnel systems of Korean companies is changing to out-group exclusivism due to changes influenced by US-style personnel systems, such as portfolio career employment. This study discusses an example of exclusivism against an outgroup by examining the relationship between proactive behavior and workplace ostracism.

This study also reveals that the higher the newcomer’s status in a new organization, the stronger the effect of workplace ostracism leading to turnover intention. Few studies have focused on the status of the victims of workplace aggression [22]. As an exception, ref. [86] studied workplace gossips and found that the lower the employee’s social status in the group’s informal network, the higher the chance of becoming a target of negative rumors. Ref. [59] predicted that new and lower-ranked members are more likely to be subject to workplace ostracism. However, these studies are related to the status and targets of workplace ostracism and present no corroborative evidence to demonstrate which groups among low- and high-ranked employees are subjected to more significant damage due to workplace ostracism.

We demonstrate that the higher the status of the newcomer, the more significant the damage caused by workplace ostracism. The effect was higher for informal status (decision-making influence) than for formal status (job rank). There seem to be two reasons for this result. First, the distinguishability among job rank has been reduced due to the personnel reform ongoing in Korean companies since the 2000s (i.e., simplification of job ranks). Second, the power (e.g., mobilization of resources, influence on other employees, and centralization in the internal communication network) indicated by informal status is more important than the formal status. Ref. [26] focus on the functional aspects of workplace ostracism and examine the possibility that the negative effect of workplace ostracism may be just as powerful for low-ranking individuals with no power in the organization, because workplace ostracism’s resource-deprivation effect is greater for employees with higher task interdependence and fewer years of service. Contrary to [64], we demonstrated that the higher the target’s status, the greater the negative effect of workplace ostracism. 

### 5.2. Practical Contributions

Our study provides some meaningful guidelines for the management of PCWs. 

First, companies should recognize that the extra-role proactive behavior of PCWs causes conflict with existing permanent employees and should help minimize the friction accompanying those behaviors. PCWs seem to understand that being critical of the workplace is dangerous. For example, according to practical wisdom, until PCWs establish a good relationship with the existing employees, they avoid demonstrating their experience gained from their previous organization, and they do not criticize the current practices even when they feel the need to present an opinion. However, as is confirmed by this study, the two types of proactive behavior are interrelated. This probably occurs because it is difficult for PCWs, who are not familiar with workplace practices, to distinguish the boundary between supportive and challenging behavior. Therefore, according to practical wisdom, the proactive behavior of PCWs may be suppressed. This is a loss for companies trying to exploit the proactive behavior of PCWs as a managerial resource.

This study proposes that a manager should conduct an administrative intervention. Three solutions can be proposed in connection with the three ostracism motives we have discussed.

First, regarding workplace ostracism used as a disciplinary measure against norm violators, managers may seem to ignore the existing norms and rules regarding the extra-role proactive behavior of PCWs and thus reduce the effectiveness of the organization. Ultimately, norm violation is interpreted at the cognitive level, and the protection of managers, who are most familiar with the conventional norms, plays an important role in recognizing norm violation.

Next, regarding workplace ostracism as a means of avoiding conflicts, managers need to create a workplace climate that does not suppress dissatisfaction or complaints. Korean companies have a collectivist culture and consider it taboo to publicize problems in the workplace. Such a cultural context enhances the necessity of workplace ostracism. Ref. [26] argue that formal policies for workplace complaints and dissatisfactions reduce workplace ostracism. 

Regarding workplace ostracism as a means of personal gain, managers need to ensure that the result of extra-role proactive behavior is not a win–lose but a win–win. Therefore, negotiations with managers and the HR department will be necessary, because the formation of win–lose relationships in Korean companies has been influenced by personnel reforms such as the introduction of a performance-based pay system in the 2000s. Korean companies had an authoritarian and paternalistic culture, and many managers evaded the new system by giving a merit rating of “A” for most of the employees to avoid having the existing order damaged by employees’ personal achievements. The HR departments at headquarters, who noticed this move, changed the performance evaluation from an absolute valuation to a relative valuation in order to prevent the performance-based pay system from becoming a mere facade. These changes intensified workplace competition and led to win–lose relationships. However, if the entire team or department’s performance improves through extra-role proactive behavior, an absolute evaluation should be considered.

Second, companies should recognize that the relationship between proactive behavior and workplace ostracism is stronger in a social exchange relationship than in an economic exchange relationship and that it is necessary to understand the context of their portfolio career employment. For example, if portfolio career recruitment is adopted to fill vacant seats caused by restructuring or employment adjustment, this recruitment expands in an economic exchange relationship. On the other hand, if portfolio career recruitment is adopted to establish a new division and rejuvenate the organizational atmosphere while retaining long-term employment practices and a seniority-based wage system, it will expand in a social exchange relationship. This study shows that “competition and conflict between PCWs and existing employees” [3] is more likely to occur in the latter case than in the former. 

Third, companies must recognize that workplace ostracism faced by high-ranked PCWs leads to turnover intention. Firms must strengthen their monitoring of PCWs hired as core talent. It is generally assumed that interpersonal workplace problems affect only new graduates and PCWs above the managerial level do not require such support. However, some PCWs are vulnerable in terms of their personal relationship within the company. In particular, the more competent a “promising” PCW is, the higher the likelihood of turnover. Therefore, HR departments must coordinate with managers and jointly determine whether PCWs hired as core personnel are facing any problems such as the silent treatment.

### 5.3. Limitations of Research and Future Directions

Despite its theoretical and practical contributions, this study has several limitations.

First, as with all studies using a cross-sectional research design, the reverse of the cause-and-effect relationship found in this study is plausible: workplace ostracism may influence proactive behavior, contrary to the hypothesis. One posits that ostracism adversely affects various attitudinal variables concerning the organization and consequently reduces employee’s cooperative behavior for the organization. This effect is found in [60], for example, who seek to clarify ostracism’s effect on organizational citizenship behavior. The other view posits that victims will increase their cooperative behavior for the organization and be much more organized due to the corrective functions of ostracism. Ref. [87], for example, performed a representative study on ostracism’s effect on prosocial behavior. If the findings of this study are understood to indicate the reverse of a cause-and-effect relationship, the negative relationship between in-role proactive behavior and workplace ostracism supports the former position, and the positive relationship between extra-role proactive behavior and workplace ostracism supports the latter position.

Second, this study measures all the variables for PCWs and thus has potential common-method problems. The logic of our hypotheses could be analyzed more concretely and the common method problem could be avoided if it were possible to gather data from both perpetrators and victims belonging to the same workplace where PCWs work together. We suggest that the relationship between proactive behavior and workplace ostracism will strengthen if (1) the unethical pro-organizational behavior of existing permanent employees is stronger, (2) the conflict avoidance behavior of existing permanent employees is stronger, and (3) the opportunistic behavior of existing permanent employees is stronger. If such a research design is adopted, the discriminatory effects exerted on workplace ostracism due to the three behaviors of existing employees can be compared. 

Third, two types of proactive behavior are compared, and their opposing effects on workplace ostracism are verified, following [33]. However, two types of attributes are superimposed on extra-role and challenging behavior and in-role and supportive behavior. If our analysis is valid, the combination of the two attributes of extra-role and challenging behavior will have a positive effect on workplace ostracism, and the combination of the two attributes of in-role and supportive behavior will have a negative effect on workplace ostracism. However, not all challenging behaviors will occur as extra-role behaviors, and not all supportive behaviors will occur as in-role behaviors. For example, challenging behavior has attributes of in-role behavior if the role draws on that authority and accelerates change within the organization (such as a task force on organizational changes).

### 5.4. Conclusions

Despite its limitations, this study clarifies how the proactive behavior of PCWs drawn from the external labor market affects their turnover intention from the standpoint of workplace ostracism. The findings could apply to all employees from the external labor market (e.g., temporary, contract, or foreign). The findings may even apply in Japan, where insiders from the internal labor market still compose the majority despite recent changes toward the external labor market. This study suggests that a rift may appear between Korean permanent employees, mainly from the perspective of organizational behavior theory, and suggests the need for complementary analyses through structural explanations such as human resources management and employment relationship theory. In other words, the workplace conflicts PCWs face not only represent interpersonal problems within the workplace, but also constitute a multilayered phenomenon related to the long-term relationships between companies and employees and the strengthened position of outgroups with new potential.

## Figures and Tables

**Table 1:** Results of confirmatory factor analysis.

Model	χ^2^	df	χ^2^/df	SRMR	RMSEA	CFI
7-factor (Model 1)	942.19	444	2.122	0.045	0.066	0.94
6-factor (Model 2)	1019.55	450	2.266	0.05	0.07	0.931
6-factor (Model 3)	1696.23	450	3.769	0.134	0.103	0.85
6-factor (Model 4)	1753.81	450	3.897	0.095	0.106	0.843
5-factor (Model 5)	1516.77	455	3.334	0.068	0.095	0.872
5-factor (Model 6)	3507.97	455	7.71	0.249	0.161	0.632
5-factor (Model 7)	2599.48	455	5.713	0.178	0.135	0.741
5-factor (Model 8)	1768.87	455	3.888	0.18	0.105	0.842
5-factor (Model 9)	1829.3	455	4.02	0.097	0.108	0.834
1-factor (Model 10)	8785.82	464	12.658	0.222	0.212	0.347

Notes. Model 1: each of the seven variables (see Appendix A) loaded on an independent factor. Model 2: extra-role proactive behavior and in-role proactive behavior loaded on a single factor. Model 3: formal job rank and decision-making influence loaded on a single factor. Model 4: workplace ostracism and turnover intention loaded on a single factor. Model 5: extra-role proactive behavior, in-role proactive behavior, and decision-making influence loaded on a single factor. Model 6: decision-making influence, formal job rank, and employee–organization relationship in HR practices loaded on a single factor. Model 7: decision-making influence, workplace ostracism, and turnover intention loaded on a single factor. Model 8: extra-role proactive behavior and in-role proactive behavior loaded on one factor, while formal job rank and decision-making influence are loaded on the other factor. Model 9: extra-role proactive behavior and in-role proactive behavior loaded on one factor, while workplace ostracism and turnover intention are loaded on the other factor. Model 10: seven variables loaded on a single factor.

**Table 2:** Means, standard deviations, and correlation coefficients.

Variables	M	SD	1	2	3	4	5	6	7	8	9	10	11	12
1. Gender (1 = male)	0.60	0.49	1											
2. Age	34.3	4.99	0.23 ***	1										
3. Tenure	2.17	0.71	0.14 *	0.15 *	1									
4. Education level	2.94	0.69	0.13 *	−0.06	0.06	1								
5. Salary (logged)	8.17	0.35	0.36 ***	0.29 ***	0.16 *	0.39 ***	1							
6. Extra-role proactive behavior	4.56	0.87	0.05	−0.05	−0.03	0.04	0.04	1						
7. In-role proactive behavior	4.39	0.78	−0.06	−0.06	−0.04	0.02	0.00	0.84 ***	1					
8. Workplace ostracism	3.02	1.36	0.15 *	−0.14 *	0.07	−0.03	0.05	−0.05	−0.25 ***	1				
9. Perceived social exchange relationship in a new organization	4.23	1.13	0.06	−0.08	0.02	0.03	0.01	0.54 ***	0.49 ***	0.02	1			
10. Formal job rank	2.01	0.72	0.39 ***	0.40 ***	0.29 ***	0.30 ***	0.61 ***	0.18 **	0.11 ^†^	−0.02	0.04	1		
11. Decision-making influence	4.44	0.98	0.09	−0.08	0.03	0.16 *	0.16 **	0.55 ***	0.52 ***	−0.04	0.41 ***	0.39 ***	1	
12. Turnover intention	3.71	1.33	−0.06	−0.08	0.05	0.03	−0.10	−0.12 *	−0.20 **	0.58 ***	−0.25 ***	−0.25 ***	−0.12 ^†^	1

Notes. Formal job rank: senior staff/assistant manager = 1, supervisor/manager = 2, assistant general manager or equivalent = 3). Tenure: 6 months–1 year = 1, less than 3 years = 2, less than 5 years = 3. Education: high school = 1, college = 2, undergraduate = 3, graduate = 4. ^†^ *p* < 0.1, * *p* < 0.05, ** *p* < 0.01, *** *p* < 0.001.

**Table 3:** Results of multiple regression analyses.

	Workplace Ostracism	Workplace Ostracism	Turnover Intention	Turnover Intention	Turnover Intention	Turnover Intention
Control variable						
Gender (1 = male)	0.132 *	0.106 ^†^	−0.067	−0.152 **	−0.129 *	−0.161 **
Age	−0.235 ***	−0.211 ***	−0.065	0.086	0.100 ^†^	0.074
Tenure	0.063	0.035	0.080	0.040	0.063	0.049
Education level	−0.089	−0.073	0.074	0.132 *	0.148 **	0.137 *
Size	−0.037	−0.070	−0.152 *	−0.128 *	−0.138 *	−0.142 **
Salary (logged)	0.113	0.120 ^†^	−0.065	−0.138 *	−0.098	−0.112 ^†^
Industrial dummy	Included	Included	Included	Included	Included	Included
Occupational dummy	Included	Included	Included	Included	Included	Included
Independent variables						
Extra-role proactive behavior	0.457 ***	0.092	0.141	−0.154	−0.123	−0.121
In-role proactive behavior	−0.649 ***	−0.658 ***	−0.308 **	0.110	0.086	0.092
Workplace ostracism				0.645 ***	0.631 ***	0.613 ***
Moderators						
Social exchange relationship		0.169 **				
Senior staff/assistant manager dummy					0.006	
Deputy general manager dummy					−0.068	
Decision-making influence						−0.031
Interactions						
Extra-role proactive behavior × social exchange dummy		0.250 *				
In-role proactive behavior × social exchange dummy		−0.236 *				
Workplace ostracism × senior staff/assistant manager dummy					−0.011	
Workplace ostracism × deputy general manager dummy					0.084 ^†^	
Workplace ostracism × decision-making influence						0.129 *
R^2^	0.243	0.279	0.114	0.429	0.439	0.446
df1, df2	18, 242	20, 240	17, 243	18, 242	20, 240	20, 240
F	4.32 ***	4.64 ***	1.84 *	10.12 ***	9.38 ***	9.66 ***
Number of respondents	261	261	261	261	261	261

Notes. Tenure: 6 months–1 year = 1, less than 3 years = 2, less than 5 years = 3. Education: high school = 1, college = 2, undergraduate = 3, graduate = 4. Standardized regression coefficients are presented (^†^ *p* < 0.1, * *p* < 0.05, ** *p* < 0.01, *** *p* < 0.001).

**Table 4:** Summary of indirect effects using bootstrapping method.

Mediated Path	Indirect Effect	Direct Effect	Total Effect
extra-role proactive behavior to turnover intention via workplace ostracism	0.439	−0.127	0.242
	**[0.236, 0.648]**	[−0.407, 0.152]	[−0.079, 0.562]
in-role proactive behavior to turnover intention via workplace ostracism	−0.607	0.09	−0.518
	**[−0.840, −0.394]**	[−0.202, 0.383]	**[−0.852, −0.183]**
extra-role proactive behavior to turnover intention via workplace ostracism (economic exchange relationship) ^1^	0.066		
	[−0.281, 0.378]		
extra-role proactive behavior to turnover intention via workplace ostracism (social exchange relationship) ^1^	0.595		
	**[0.338, 0.883]**		
in-role proactive behavior to turnover intention via workplace ostracism (economic exchange relationship) ^1^	−0.314		
	[−0.634, 0.026]		
in-role proactive behavior to turnover intention via workplace ostracism (social exchange relationship) ^1^	−0.832		
	**[−1.101, −0.579]**		
extra-role proactive behavior to turnover intention via workplace ostracism (“senior staff/assistant manager or equivalent”) ^2^	0.363		
	**[0.202, 0.535]**		
extra-role proactive behavior to turnover intention via workplace ostracism (“supervisor/manager or equivalent”) ^2^	0.43		
	**[0.266, 0.604]**		
extra-role proactive behavior to turnover intention via workplace ostracism (“deputy general manager or equivalent”) ^2^	0.497		
	**[0.305, 0.698]**		
in-role proactive behavior to turnover intention via workplace ostracism (“senior staff/assistant manager or equivalent”) ^2^	−0.535		
	**[−0.862, −0.223]**		
in-role proactive behavior to turnover intention via workplace ostracism (“supervisor/manager or equivalent”) ^2^	−0.561		
	**[−0.803, −0.334]**		
in-role proactive behavior to turnover intention via workplace ostracism (“deputy general manager or equivalent”) ^2^	−0.761		
	**[−1.065, −0.477]**		
extra-role proactive behavior to turnover intention via workplace ostracism (low level of decision-making influence) ^2^	0.34		
	**[0.156, 0.544]**		
extra-role proactive behavior to turnover intention via workplace ostracism (high level of decision-making influence) ^2^	0.498		
	**[0.277, 0.728]**		
in-role proactive behavior to turnover intention via workplace ostracism (low level of decision-making influence) ^2^	−0.477		
	**[−0.751, −0.243]**		
in-role proactive behavior to turnover intention via workplace ostracism (high level of decision-making influence) ^2^	−0.698		
	**[−0.952, −0.450]**		

Notes. **Bold** indicates that 95% confidence interval excludes zero. *n* = 261. ^1^ denotes a first-stage moderated mediation model, and ^2^ denotes second-stage moderated mediation model.

## Data Availability

The data presented in this study are available on request from the corresponding author. The data are not publicly available due to respondent’s permission was not acquired.

## Appendix A

**[Proactive Behavior]**

Extra-role proactive behavior (“voice behavior”)

1. I speak up and encourage others in this group to get involved in issues that affect the group.
2. I communicate my opinions about work issues to others in this group even if my opinion is different and others in this group disagree with me.
3. I keep well-informed about issues where my opinion might be useful to this work group.
4. I get involved in issues that affect the quality of work life here in this group.
5. I speak up in this group with ideas for new projects or changes in procedures.

In-role proactive behavior (“continuous improvement”)

1. I devise ways to improve processes.
2. I seek to increase productivity and quality.
3. I gather and utilize available information.
4. I am inquisitive and proactive.

Workplace ostracism

1. Others ignored me at work.
2. Others left the area when I entered.
3. My greetings have gone unanswered at work.
4. Others at work treated me as if I wasn’t there.
5. Others refused to talk to me at work.
6. I have been included in conservation at work (reverse coded).

Turnover intention

1. I tend to leave this organization soon.
2. I plan to leave this organization in the next little while.
3. I will quit this organization as soon as possible.
4. I do not plan on leaving this organization soon (reverse coded).
5. I may leave this organization before too long.

Perceived social exchange relationship in a new organization

1. This organization evaluates employees based on unit performance.
2. This organization rewards employees based on unit performance.
3. This organization assigns performance goals or standards that focus on the employee’s work group or unit.
4. This organization trains employees in skills that prepare them for future jobs and career development.
5. This organization provides career counseling and planning assistance to employees.
6. This organization provides employees with employment security.
7. This organization recruits employees from within the organization.

**[Status in a new organization]**

Formal job rank (single item)
senior staff/assistant manager or equivalent;
supervisor/manager or equivalent;
deputy general manager or equivalent
Decision-making influence

1. I influence decisions about ways to improve productivity.
2. I influence decisions about ways to improve the quality of the work environment.
3. I influence decisions about ways to improve the quality of the product or service.
4. Overall, I have influence on decision-making processes in this organization.
